# Synthetic sugar cassettes for the efficient production of flavonol glycosides in *Escherichia coli*

**DOI:** 10.1186/s12934-015-0261-1

**Published:** 2015-06-09

**Authors:** Prakash Parajuli, Ramesh Prasad Pandey, Nguyen Thi Huyen Trang, Amit Kumar Chaudhary, Jae Kyung Sohng

**Affiliations:** Department of BT-Convergent Pharmaceutical Engineering, Institute of Biomolecule Reconstruction, Sun Moon University, 70 Sunmoon-ro 221, Tangjeong-myeon, Asan-Si, Chungnam 336-708 Republic of Korea

**Keywords:** NDP-sugars biosynthesis circuits, Glycosyltransferase, Multi-monocistronic vector, Flavonol glycosides

## Abstract

**Background:**

A multi-monocistronic synthetic vector was used to assemble multiple genes of a nucleotide diphosphate (NDP)-sugar biosynthetic pathway to construct robust genetic circuits for the production of valuable flavonoid glycosides in *Escherichia coli*. Characterized functional genes involved in the biosynthesis of uridine diphosphate (UDP)-glucose and thymidine diphosphate (TDP)-rhamnose from various microbial sources along with glucose facilitator diffusion protein (*glf*) and glucokinase (*glk*) from *Zymomonas mobilis* were assembled and overexpressed in a single synthetic multi-monocistronic operon.

**Results:**

The newly generated NDP-sugars biosynthesis circuits along with regiospecific glycosyltransferases from plants were introduced in *E. coli* BL21 (DE3) to probe the bioconversion of fisetin, a medicinally important polyphenol produced by various plants. As a result, approximately 1.178 g of fisetin 3-*O*-glucoside and 1.026 g of fisetin 3-*O*-rhamnoside were produced in UDP-glucose and TDP-rhamnose biosynthesis systems respectively, after 48 h of incubation in 3 L fermentor while supplementing 0.9 g of fisetin. These yields of fisetin glycosides represent ~99% of bioconversion of exogenously supplemented fisetin. The systems were also found to be highly effective in bio-transforming other flavonols (quercetin, kaempferol, myricetin) into their respective glycosides, achieving over 95% substrate conversion.

**Conclusion:**

The construction of a synthetic expression vector for bacterial cell factory followed by subsequent re-direction of metabolic flux towards desirable products have always been revolutionized the biotechnological processes and technologies. This multi-monocistronic synthetic vector in a microbial platform is customizable to defined task and would certainly be useful for applications in producing and modifying such therapeutically valued plant secondary metabolites.

**Electronic supplementary material:**

The online version of this article (doi:10.1186/s12934-015-0261-1) contains supplementary material, which is available to authorized users.

## Background

The bioactive flavonoids are considered as the most important phytochemicals abundantly present in daily dietary foods and are of great interest due to their diverse health beneficial activities for the management of chronic diseases [1]. Flavonols are a class of flavonoids having 3-hydroxyflavone backbone and different stems hydroxylated by phenolic –OH groups to characterize as quercetin, kaempferol, myricetin, morin and fisetin [2]. Being the most active compounds within flavonoid sub-group, they exhibit wide range of biological activities such as prevention of cardiovascular diseases [3, 4], antioxidant [5], anti-diabetic activity [6], anticancer activity [7], etc. Although flavonols are virtually present in almost all the vegetables and fruits, they are usually present in diverse glycoside forms with considerable amounts in our normal diet [3]. Glycosides are produced through an approach called glycosylation which is considered to be a key modification in plant secondary metabolites and it is also one of the major factor that determines natural products solubility, bioactivity and bioavailability [8]. Additionally, most flavonoid drugs (quercetin 3-*O*-rutinoside, daidzein 8-C-glucoside) presently in clinical applications are in the form of glycosides [1]. So these significance have attracted considerable interest in current research trend to produce them rationally in industrial scale. Microbial biotransformation is one of the biotechnological approach for the production of such bioactive flavonoids and their glycosides in desirable scale [1, 9]. Biotransformation of flavonoids could be achieved from many microorganisms including species of *Aspergillus*, *Bacillus*, *Saccharomyces*, *Streptomyces*, *Escherichia coli* [1, 10].

*Escherichia coli* is one of the industrially important microorganisms, widely used in different research facilities for the production of therapeutic proteins [11], bio-based chemicals and bio-fuel production [12], production and modifications of natural products [13, 14], production of valuable therapeutics and cosmetics [15–18], neutraceuticals [18, 19], etc. *E. coli* is often possible to grow in high cell densities by utilizing simple carbon sources and dissolve oxygen to accomplish volumetric productivity [20]. Additionally, recently developed synthetic biology approaches helped to establish *E. coli* based efficient cell factories by genetic manipulations or recombining foreign genetic materials for efficient utilization of simple precursors with high growth rate, avoiding metabolic shutdown [21].

In this aspect, several flavonols have been biotransformed to produce their glycosides using different metabolic or combinatorial approaches and synthesized compounds with high stereo- and regio-selectively [22]. Overexpression of a single glycosyltransferase in wild type host strain and whole-cell biotransformation of flavonoids to glycosides would be an easy approach. But, construction of a cell factory through synthetic approaches (enzyme engineering, knockdown and knockout for metabolic flux control, heterologous expression of pathway specific genes) are the key parameters basically involved while designing high yield production strains [23]. Application of synthetic vector and heterologous expression of multiple genes into single vector has been one of the alternatives while talking about an efficient cell factory construction and synthesis of modified secondary metabolites [24, 25]. Additionally, metabolic engineering approaches have been applied by various groups to synthesize diverse glycosides of flavonols using *E. coli* biotransformation systems.

In the current experiment, the multi-monocistronic vector piBR181 [26] was used as a vehicle to construct the sugar cassettes and employ them for the glycosylation of flavonols. For the production of high value flavonoid glycosides, nucleotide diphosphate (NDP)-sugar biosynthesis specific genes were chosen and assembled in a single vector along with glucose facilitator diffusion proteins (*glf* and *glk*) from *Zymomonas mobilis* [27, 28]. To facilitate the synthesis of flavonol glucosides and rhamnosides, regiospecific glycosyltransferases (GTs), specifically, uridine diphosphate (UDP)-3-*O*-glycosyltransferase (UGT78K1) from *Glycine max* and flavonols-3-*O*-rhamnosyltransferase (ArGt-3) from *Arabidopsis thaliana* were selected. Additionally, consumption of glucose as the sole carbon source was optimized in the *E. coli* strain and its subsequent utilization by the facilitator proteins for accelerating the production of glycosides was studied. These single vector NDP-sugar biosynthesis systems were compared with previously reported multi vector biotransformation systems [29–31] for increased titer of flavonoid glycosides under identical conditions.

## Results and discussion

### Construction of NDP-sugar biosynthesis systems

**Figure 1:**
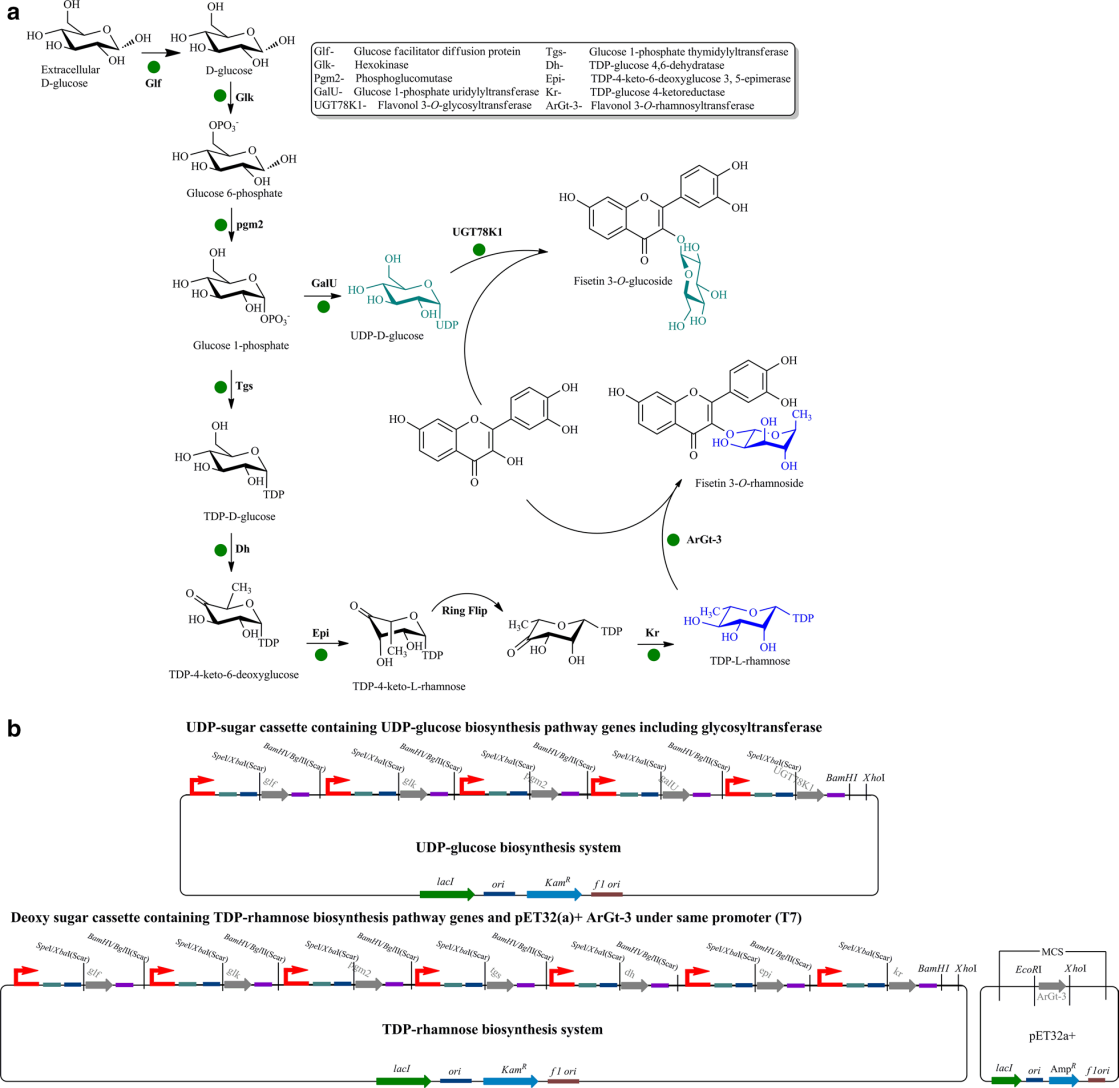
**a** NDP-sugar (UDP-d-glucose and TDP-l-rhamnose) biosynthesis pathway in *E. coli* showing the pathway overexpressed genes and glycosyltransferases (*green dots*). **b** Newly constructed sugar cassettes for the biosynthesis of TDP-rhamnose and UDP-glucose using a multi-monocistronic vector piBR181.

NDP-sugar biosynthesis genes from different source organisms were individually cloned and assembled to construct UDP-glucose and thymidine diphosphate (TDP)-rhamnose sugar cassettes in piBR181 vector containing multi-monocistronic operon systems [26]. Five different UDP-glucose pathway genes including glucose facilitator diffusion protein and flavonol-3-*O*-glycosyltransferase were cloned successfully to construct a UDP-glucose biosynthesis system. Seven TDP-l-rhamnose biosynthesis genes, including facilitator diffusion protein, were cloned and assembled to construct TDP-rhamnose sugar cassettes (Figure 1). Sources and cloning strategies for each gene are explained in materials and methods. The vector physical map of both sugar cassettes and assembly of genes are given in supplementary data (Additional file 1: Figures S1, S2).

The UDP-glucose system consists of three UDP-glucose biosynthesis genes: glucokinase (*glk*; PCR size 984 kb) which catalyzes the addition of phosphate groups into the 6-hydroxyl position of d-glucose immediately after entry into the cell, phosphoglucomutase (*pgm2*; 1,749 kb) which synthesizes glucose 1-phosphate from glucose-6-phosphate, and glucose 1-phosphate uridylyltransferase (*galU*; 908 kb) which transfers the uridine diphosphate (UDP) group to make UDP-glucose. In addition to the above three genes, glucose facilitator diffusion protein (*glf*; 1,422 kb), which helps in the internalization of extracellular glucose present in the medium into the cell to increase the pool of UDP-glucose and the regiospecific flavonol 3-*O*-glycosyltransferase (*UGT78K1*; 1,344 bp), which catalyzes the transfer of glucose moiety from activated UDP-glucose into the acceptor flavonols at the 3-hydroxyl position, were also assembled in the same vector to generate a 7,312 bp UDP-glucose system. This UDP-glucose cassette was transferred into *E. coli* BL21 (DE3) for further biotransformation experiments. Similarly, the same *glf*, *glk*, and *pgm2* genes were recombined along with glucose 1-phosphate thymidylyltransferase (*tgs*; 882 bp, synthesizes TDP-glucose from glucose-1-phosphate and thymidine triphosphate), and TDP-glucose 4, 6-dehydratase (*dh*; 1,086 bp) to make TDP-4-keto-6-deoxyglucose intermediate. The modifying genes for this intermediate, TDP-4-keto-6-deoxyglucose 3, 5-epimerase (*ep*; 609 bp) and TDP-glucose 4-ketoreductase (*kr*; 885 bp), were also cloned to the same vector to complete the 8,884 bp TDP-rhamnose biosynthesis system. A regiospecific flavonol 3-*O*-rhamnosyltransferase (ArGt-3) was cloned separately into pET32(a) + vector to generate pET32(a) + ArGt-3 because of the complications of restriction sites. The TDP-rhamnose sugar cassette along with pET32(a) + ArGt-3 were transformed into the *E. coli* BL21 (DE3) host for biotransformation reaction.

### Bioconversion of fisetin

**Figure 2:**
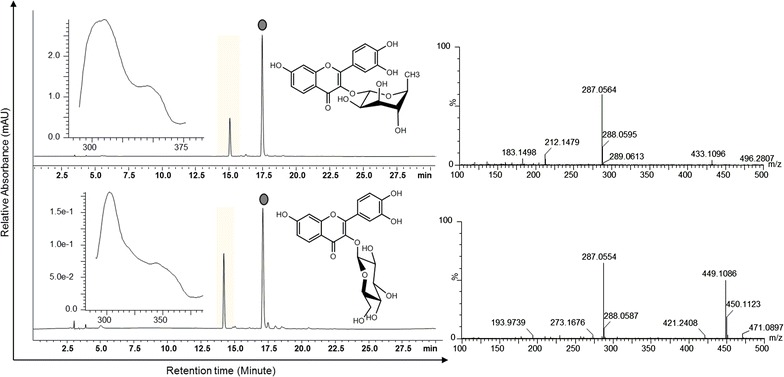
HPLC-PDA chromatogram and HR-QTOF ESI LC/MS analysis showing the production of fisetin glycosides (fisetin 3-*O*-glucoside and fisetin 3-*O*-rhamnoside) from the biotransformation of fisetin.

*E. coli* BL21 (DE3) harboring pIBR181-UGT78K1 (Strain S_1_) and pET32a(+)ArGt-3 (Strain S_5_) were used to check the bioconversion of exogenously supplemented fisetin as described in materials and methods. The high performance liquid chromatography (HPLC) chromatograms of each extract from the biotransformation reactions showed new peaks at retention time ~14.4 min for fisetin 3-*O*-glucoside and ~14.8 min for fisetin 3-*O*-rhamnoside compared to the standard fisetin detected at ~17.1 min at the UV absorbance of 320 nm (Figure 2). These product peaks detected from the glycosylation systems were further analyzed by high resolution liquid chromatography-quadrupole time-of-flight electrospray ionization mass spectrometry resolution (LC-QTOF-ESI/MS) in positive ion mode. The mass spectra displayed the exact mass of fisetin 3-*O*-rhamnoside [M + H]^+^*m*/*z*^+^ ~433.1096 and fisetin 3-*O*-glucoside [M + H]^+^*m*/*z*^+^ ~449.1086 along with the spectrum for fisetin [M + H]^+^*m*/*z*^+^ ~287.0564 (Figure 2). The control experiments were carried out in identical conditions by supplementing fisetin in same strains without induction. In control strains, no bioconversion of fisetin was observed while analyzing HPLC–PDA.

**Figure 3:**
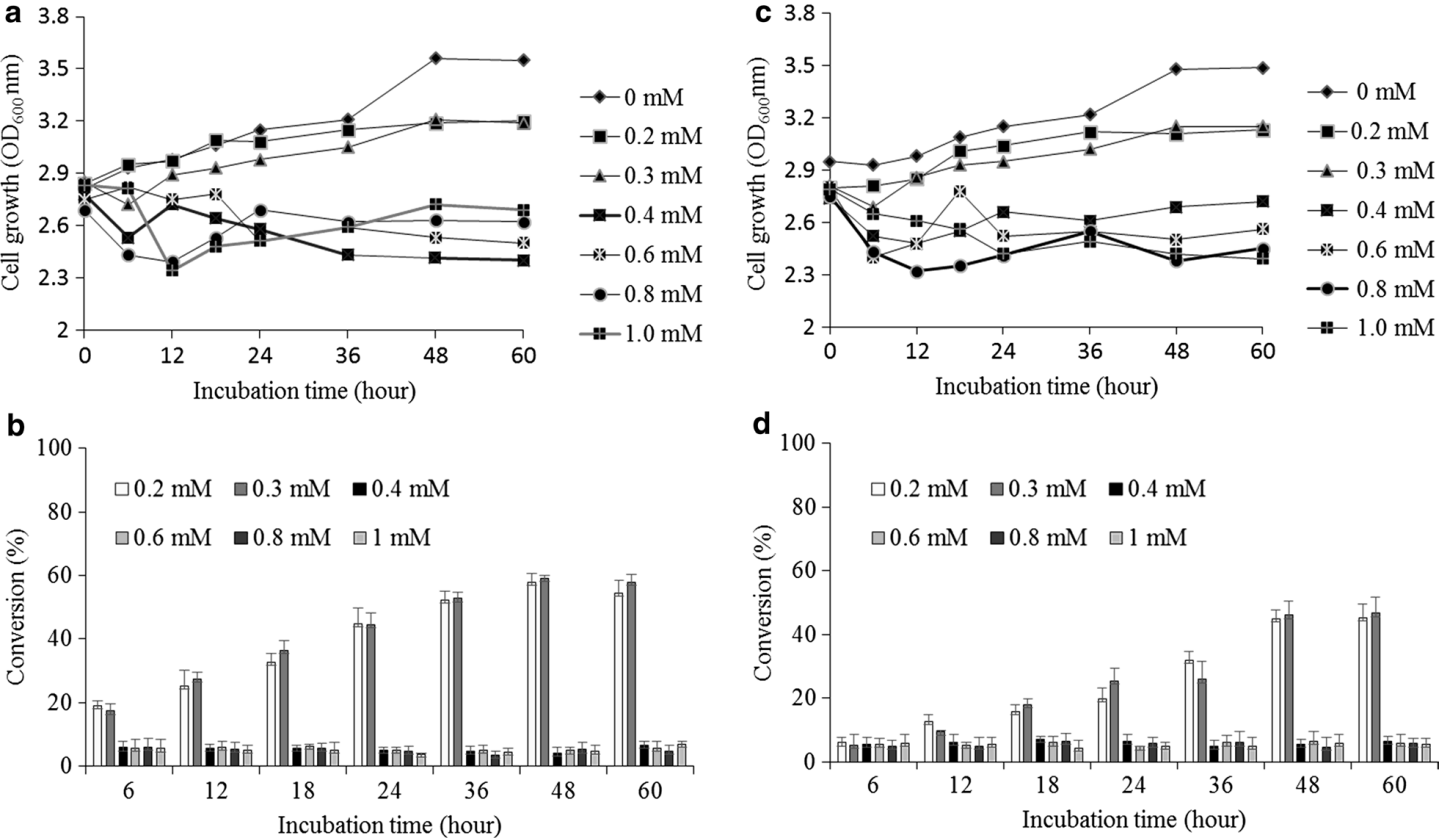
Substrate optimization from different concentration of fisetin (0.2, 0.3, 0.4, 0.6, 0.8, 1.0 mM) according to the cell growth at OD_600nm_ and production of fisetin 3-*O*-glucoside (**a**, **b**) and fisetin 3-*O*-rhamnoside (**c**, **d**) in 60 h incubation time by the strains S_1_ and S_5_, respectively.

To study the substrate inhibition for the biotransformation, different concentrations (0.2, 0.3, 0.4, 0.6, 0.8, 1.0 mM) of fisetin were fed into biocatalyses reaction systems (strains S_1_ and S_5_). The cell growth and substrate conversion were monitored in both shake flask cultures (50 mL) for 60 h as explained in materials and methods. The bioconversion of fisetin into its glycoside were exponentially increased until 48 h and become static for 60 h. So, we calculated the total conversion based on 48 h incubation. The maximum bioconversion of fisetin recorded was 58.9% in strain S_1_ and 41.8% in strain S_5_ while supplementing 0.3 mM of fisetin at the highest cellular growth of OD_600_ ~3.5 for 48 h. Upon increasing the concentration of fisetin from 0.3 mM, both strains showed relatively lower substrate conversion with decreased cell growth rates (Figure 3). The results suspected were toxic environment due to high concentration fisetin in *E. coli* culture medium [33].

### Glucose utilization and its impact on improved bioconversion by glucose facilitator protein

**Figure 4:**
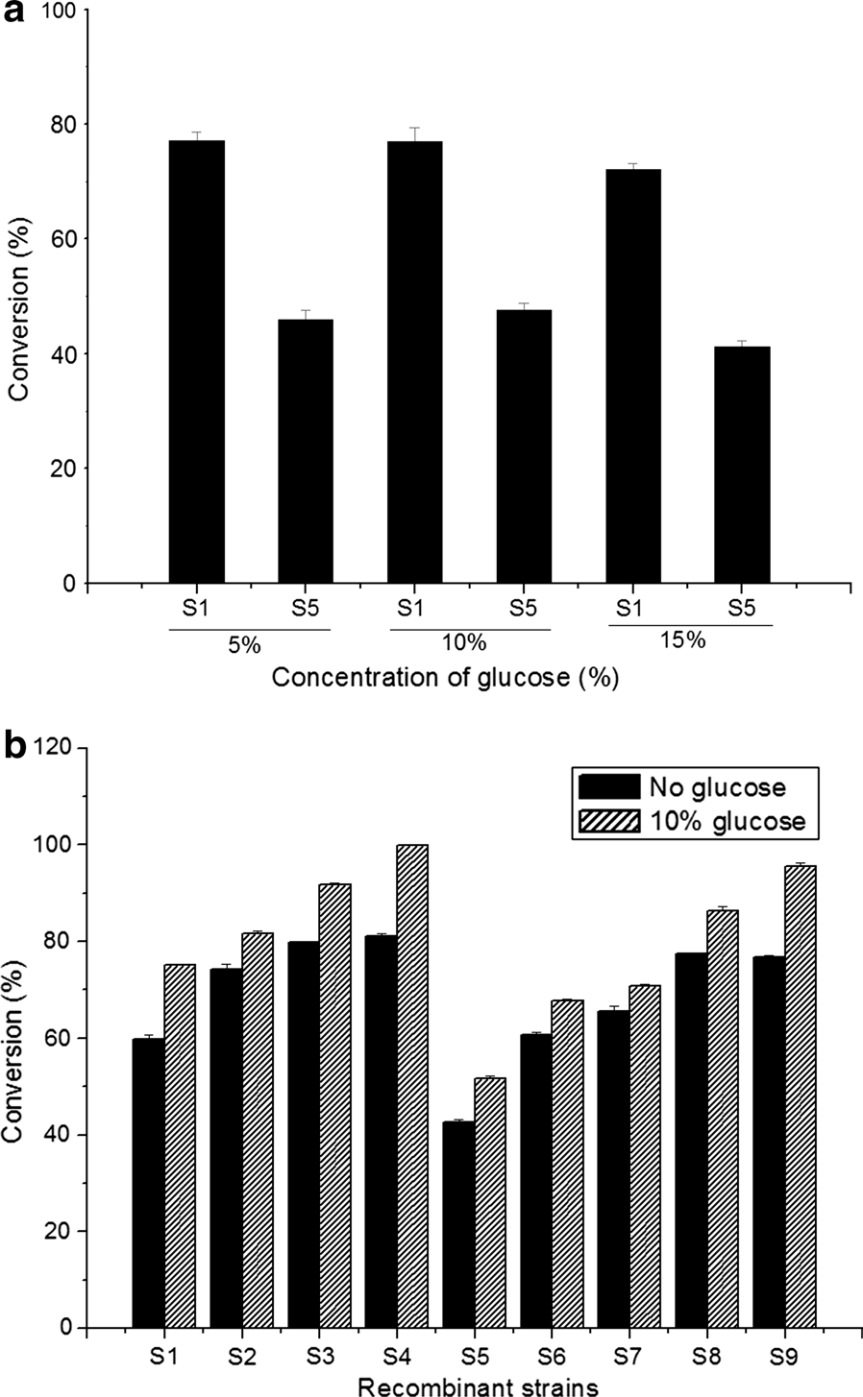
**a** Optimization of glucose concentration based on the recombinant strain S_1_ and S_5_ (S_1_ and S_5_ contains only GTs UGT78K1 and ArGt-3, respectively) in 48 h incubation. Maximum conversion of fisetin to respective glycosides was achieved while supplementing 10% additional glucose in the medium. **b** Production profile of respective glycosides in all the constructed recombinant strains (S_1_–S_9_) in optimized concentration of substrate (0.3 mM) and glucose (10%) in 48 h incubation time. Maximum conversion of fisetin to fisetin 3-*O*-glucoside was achieved to be ~100%. The conversion of fisetin to fisetin 3-*O*-rhamnoside was ~96%.

Three different concentrations (5, 10, 15%) of glucose were supplemented in separate cultures of strains S_1_ and S_5_. The utilization of glucose by these strains was assessed by the biotransformation reactions of exogenously fed 0.3 mM fisetin in HPLC–PDA. The HPLC–PDA analysis found that 77.1% of fisetin was converted to fisetin-3-*O*-glucoside whereas the conversion of fisetin to fisetin 3-*O*-rhamnoside was limited to 48.5% in strains S_1_ and S_5_, respectively, upon addition of 10% additional glucose to the medium over 48 h of incubation (Figure 4a). The supplementation of 10% glucose and 0.3 mM fisetin acceptor substrate was optimized for utilization by the *E. coli* strains, balancing their metabolic flux and physiology in respect to the cellular growth and product formation.

The effects of supplementing extra 10% glucose in the medium and over expression of glucose facilitator protein on bioconversion of fisetin were also evaluated by feeding 0.3 mM of fisetin in various strains. The recombinant strains harboring multi-vectors overexpressing UDP-glucose and TDP-rhamnose pathway specific genes but without glucose facilitator protein (strains S_2_, S_6_, and S_7_), monocistronic vector carrying pathway specific genes without glucose facilitator protein (strains S_3_, S_8_) and with glucose facilitator protein (strains S_4_ and S_9_), were grown in identical conditions for biotransformation. The samples were taken at different time intervals for HPLC–PDA analysis to determine the substrate conversion rate. Regarding 3-*O*-glucoside production, strain S_3_ (mono-cistronic vector construct carrying UDP-glucose biosynthesis genes) was found to be more effective (~85% conversion) than strain S_2_ (~80% conversion) which carried multiple vectors for the UDP-glucose biosynthesis. Similar results were observed for the production of fisetin 3-*O*-rhamnoside. Strain S_6_ and S_7_ harboring TDP-rhamnose biosynthetic pathway genes in multiple vectors exhibited lower conversion (~68, ~72%, respectively) than strain S_8_ (~80%) in which those genes were cloned in mono-cistronic vector system. However, the recombinant strain S_4_ harboring a multi-monocistronic vector with sugar pathway genes for UDP-glucose glycosylation system along with glucose facilitator diffusion protein (*glf*) and 10% exogenous glucose supplement, it was able to convert ~100% substrate into product within 48 h. Similarly, for fisetin 3-*O*-rhamnoside production, the recombinant strain with multi-monocistronic TDP-rhamnose biosynthesis systems and facilitator proteins and 10% exogenous glucose supplement, it (S_9_) was able to convert ~95% of fisetin (Figure 4b).

Thus, comparing the production profile of all the recombinant strains with multi-vector and multi-monocistronic NDP-sugar biosynthesis systems, use of the multi-monocistronic vector for multiple gene expression was found to be highly effective. With respect to the acceleration of the system, glucose facilitator protein could significantly increase the production of respective glycosides by ~20% compared to strains without facilitator genes.

### Scale-up by fermentation

The recombinant strains S_4_ and S_9_ were chosen for large scale production of fisetin 3-*O*-glucoside and fisetin 3-*O*-rhamnoside respectively as both strains exhibited efficient bioconversion of fisetin (Figure 4b). Cultures of both strains were prepared for 3-L fermentation as described in materials and methods where optimized concentrations of substrate (~300 mg in 3 L (~0.35 mM)) and glucose (10%) were supplemented. The temperature of the fermentor was kept at 25°C and the pH was 7.0 during the entire period for both systems. The culture medium were harvested at a regular time interval of 12 h and analyzed by HPLC–PDA to monitor the conversion of fisetin into its glycosides. The rest of the fermentation condition was identical to our previous studies [30, 34].

**Figure 5:**
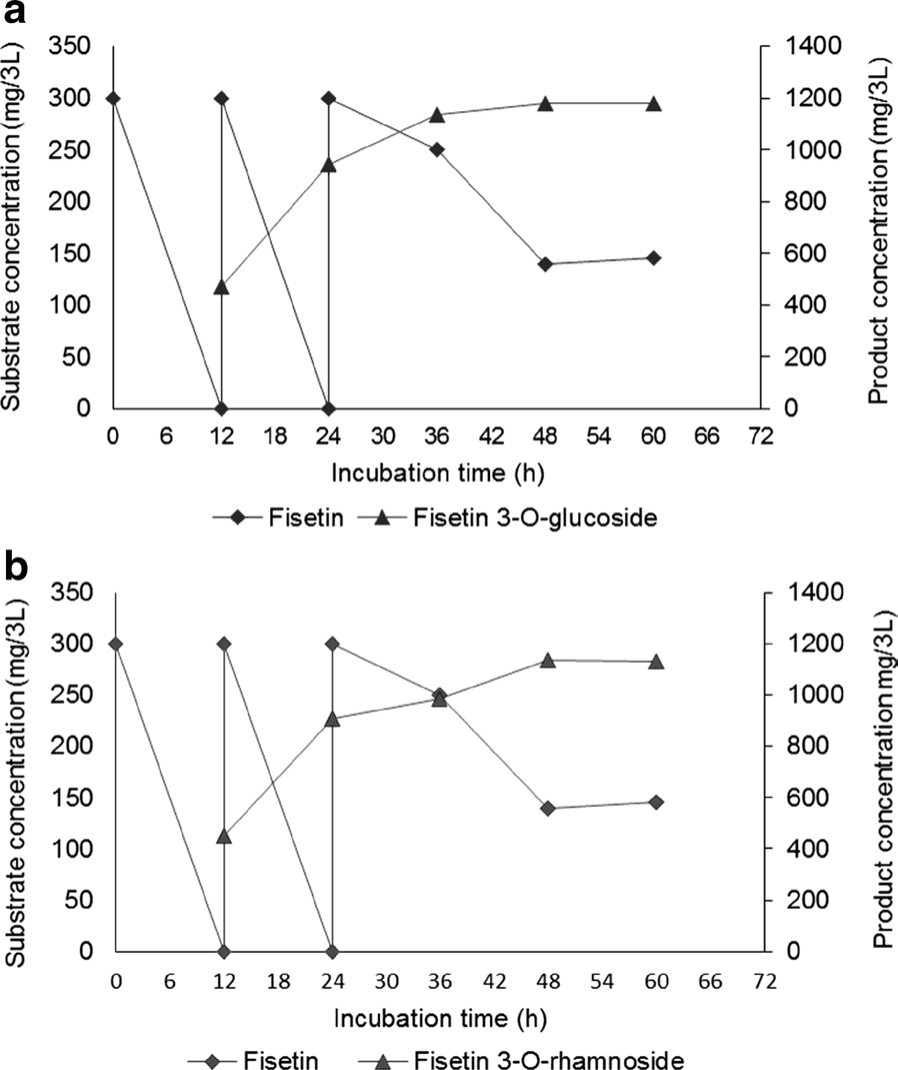
The scale-up production of fisetin glycosides in 3 L fermentation at different time intervals. **a** Production profile of fisetin 3-*O*-glucoside by strain S_4_. **b** Production profile of fisetin 3-*O*-rhamnoside by strain S_9_. 0.35 mM fisetin dissolve in DMSO was successively added three times in both 3-L fermentation systems.

The HPLC analysis of the 3-L fermentor bioconversion samples at 12 h revealed complete conversion of fisetin to fisetin 3-*O*-glucoside and fisetin 3-*O*-rhamnoside by respective strains (Figure 5). Thus, additional 300 mg of fisetin was added in 12 h and continued the fermentor under identical conditions. The HPLC samples analysis at 24 h also exhibited approximately 100% conversion of fisetin to glycosides. Hence, again the same amount of fisetin was added at 24 h and proceeded the fermentation. However, after 24 h, the bioconversion of fisetin was slower than previous in both systems. The conversion rate was almost constant from 48 to 60 h (Figure 5). The fisetin added at 24 h was not completely converted until 60 h. Overall calculations revealed the production of ~1.178 g of fisetin 3-*O*-glucoside [~393 mg/L (~0.30 mM)] and 1.026 g of fisetin 3-*O*-rhamnoside [~ 342 mg/L (~0.27 mM)] in 48 h from the 3 L fermentor from 0.9 g (~1.04 M) fisetin supplemented (Figure 5).

### Biotransformation of other flavonols

**Figure 6:**
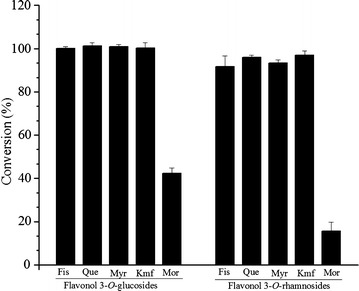
Biotransformation of other flavonols (Fis-fisetin, Que-quercetin, Myr-myricetin, Kmf-kaempferol, Mor-morin) into respective glycosides using the best recombinant strains (S_4_ and S_9_).

After successfully probing and comparing the monocistronic and multivector UDP-glucose and TDP-rhamnose biosynthesis systems for the biotransformation of fisetin as a substrate, we used the best monocistronic UDP-glucose and TDP-rhamnose biosynthesis genes and glucose facilitator gene containing systems (strains S_4_ and S_9_, respectively) to synthesize other flavonol glycosides. Kaempferol, myricetin, morin and quercetin were biotransformed into their respective glucosides and rhamnosides. The biotransformation of all the flavonols were done in a shake flask supplementing 10% glucose in culture medium along with 0.3 mM of each individual flavonoid as substrate. Similar to the fisetin, there was approximately 95–100% conversion of kaempferol, quercetin and myricetin substrates into their respective glucosides and rhamnosides. However, the conversion of morin was limited to <40% in strain S_4_ and <15% in strain S_9_ (Figure 6, Additional file 1: Figure S3). These regiospecific glucoside and rhamnoside products of other flavonols were confirmed by high resolution mass analyses (Additional file 1: Figure S4).

Thus, the overall result of the study indicates that the assembly of biological standard parts in multi-monocistronic fashion under promoters of the same strength is of great important for the production of bioactive flavonoid glycosides. This system yielded higher conversion rates than the multi-vector system reported previously. It is certain that production of the glycosides of flavonols other than morin should be similar to fisetin which we probed in the constructed system (Additional file 1: Figure S3).

## Discussion

A multi-monocistronic operon system (piBR181) was generated previously modifying the commercially available vector pET28a (+) in order to clone multiple genes under their own promoter [26]. The construct constitutes of computationally designed 181-bp ds-DNA bio-parts ligated to pET-28a (+) at the *Bgl*II and *Xho*I restriction sites replacing original one to generate expression vector piBR181. However, *Bgl*II and *BamH*I-*Xho*I restriction sites presents before the promoter and after the transcription termination sites for multi-monocistronic operon assembly. Likewise, *Spe*I-*Hind*III remained was for PCR cloning site. Large number of gene can be cloned into this vector system and their functional expression are assumed to be independent transcriptome because they have independent bio-parts (T7 promoter, Lac operator, Ribosome binding site and T7 terminator). Having multiple copy number, it is applicable for multiple gene expression in multi-copy number transcriptome so that high yield production could be achieved. Moreover, necessity of using multiple vectors for heterologous protein expression and use of various antibiotic creates stress to the culture medium can be reduced. But the limitation is, five restriction sites (*Bgl*II, *Spe*I, *Hind*III, *BamH*I and *Xho*I) must be absent from the gene sequence and vector itself.

The production of flavonoid was much higher in monocistronic operon compared to pseudo-operon where the distance between the start and end of the operon was an important factor in reducing premature transcription termination and mRNA degradation [35, 36]. Multiple circuits in an operon with the termination sequence present at every end of the gene set under the same promoter could therefore improve multi-round transcription by recycling the T_7_ RNA polymerase quickly, thereby facilitating proper folding of multiple proteins [37]. In present study also, the efficiency of multi-monocistronic sugar biosynthesis system compared to multiple vector systems was found to be remarkable for producing flavonol glycosides. Each recombinant strains harboring multi-vectors and multi-monocistronic vector constructed using UDP-glucose and TDP-rhamnose biosynthesis pathway genes were compared for production of fisetin 3-*O*-glucoside and fisetin-3-*O*-rhamnoside catalyzed by UGT78K1 and ArGt-3 with the substrate fisetin. The production of flavonoid glycosides was comparably high using multi-monocistronic constructs in this experiment (Figure 4b). In previous studies, 24 mg/L of 3-*O*-rhamnosyl quercetin and 12.9 mg/mL 3-*O*-rhamnosyl kaempferol was produced using a multiple vector system [31]. Likewise, The relative bioconversion of flavonols into rhamnosides using the same glycosyltransferases (ArGt-3), although 100% quercetin was converted into product while the other flavonols (kaempferol—85.7%, Myricetin—20.5%, Fisetin—62.3% and morin—0%) were converted by a lesser amount [38]. Similarly, Kim et al. [39] reported the maximum production of 150 mg/L of quercetin 3-*O*-rhmanoside as and 200 mg/L of kaempferol 3-*O*-rhamanoside. Similarly, 98 mg/L of quercetin 3-*O*-(6-deoxytalose) was produced through *E. coli* metabolic engineering approach [40].

Looking at the aforementioned production level of each flavonol glycosides, we could hypothesize several reasons to increase the titer more than previously reported. In most of the engineering strategy NDP-sugar biosynthesis pathway specific genes has been deleted to divert the metabolic flux for desirable sugar pool increment [31, 38, 40, 41]. But this may require additional supplementation of carbon source and limits the optimum growth as compared to wild type strain. Similarly, use of multiple vectors can affect the production level since various antibiotics in culture broth limits the cell growth [35], different rate of plasmid replication because of varying plasmid origin [42] and multiple gene expression under different promoter strength can have varied expression of genes which could ultimately reduce the production of target compounds [14, 42]. Use of synthetic biology tools by engineering each regulatory components of an expression vector itself for the assembly of multiple genes and allow them to express consecutively could address aforementioned limitations of high yield production of target compounds from microbial cells [36, 43]. So there will be no metabolic burden for cell itself because of similar strength promoter architecture and could achieve tunable gene expression in transcriptional level. Use of single vector system in multi-monocistronic fashion and assembly of multi-pathway genes has proven advantages over old strategies of constructing cell factory with multiple antibiotic resistance vectors containing pathway specific genes [44, 45]. Production of vaccine through construction of multi-gene expression cassette [46], tandem repetitive promoter in expression vector increases the expression level of genes ultimately scale up in secondary metabolite production [47], gram scale production of chondroitin [24] and catechin [25] using synthetic vectors in *E. coli* are the successful examples.

The construction of an artificial sugar cassette facilitated bacterial cell factory and subsequent re-direction of metabolic flux towards desirable products achieved significant production of flavonol 3-*O*-glucoside and 3-*O*-rhamnoside compared to previously reported data. However, further strengthening sugar biosynthesis system assembling glucose facilitator diffusion protein (Glf) with the goal of increasing the pool of intermediate product (glucose 1-phosphate) effectively increased the production of each glycoside by 20%. Consequently, the production of fisetin 3-*O*-glucoside was approximately 1.178 g/3 L-fermentation (~393 mg/L) in 48 h and 1.026 g/3 L-fermentation (~342 mg/L) of fisetin 3-*O*-rhamnoside within the same time limit. Since the conversion to both glycosides was remarkably high, these are the best strains and production yields ever reported.

The same recombinant systems were also used to synthesize other regiospecific flavonol glycosides using kaempferol, quercetin, myricetin and morin as substrates. The result demonstrated a relative product conversion rates of ~100% for quercetin, myricetin and kaempferol for 3-*O*-glucosides production while ~95% for quercetin, myricetin and kaempferol for 3-*O*-rhamnosides production in 48 h while supplementing 0.3 mM of each substrate (Figure 6). Thus, the proposed glycosylation systems could be used as model systems in the glycoconjugation of similar derivatives as pharmaceutical ingredients.

## Methods

### Microorganisms, plasmids, culture conditions and chemicals

All the strains, vectors and plasmids used in this study are listed in Table 1. Recombinant strains harboring the NDP-sugar biosynthesis genes are numbered from S_1_ to S_9_. For the selection and maintenance of plasmids, *E. coli* strains were grown at 37°C in Luria–Bertani (LB) broth or on agar plates supplemented with the appropriate amount of antibiotics (ampicillin—100 μg/mL, kanamycin—50 μg/mL, streptomycin 50 μg/mL and chloramphenicol 50 μg/mL). pGEM-T® easy vector (Madison, Promega), pET32a(+), pCDF-Duet-1, pACYC-Duet-1 (Novagen, Darmstadt, Germany) and piBR181 vectors were used for the cloning of polymerase chain reaction (PCR) products and for expression of the genes (Additional file 1: Table S1). All the chemicals (methanol, water, ethyl acetate, etc.) used in this study were purchased from either Sigma-Aldrich or another high grade commercial source. All restriction enzymes used in the cloning process were obtained from Takara (Shiga, Japan). Oligonucleotide primers were from Genotech (Daejeon, Korea). The rest of the chemicals purchased from commercially available sources were of high grade.

**Table 1:** Bacterial strains, plasmids used in this study

Vectors and plasmids	Description	Source/references
pGEM®-T easy vector	General cloning vector, T7 and SP6 promoters, f1 ori, Amp^r^	Promega, USA
pIBR181	Multi mono-cistronic vector modified from pET28a + , f1 pBR322 ori, Km^r^	Chaudhary et al. [13]
piBR181-UGT78K1	piBR181 vector carrying UGT78K1	This study
pET32(a) + ArGt-3	pET32(a) + vector carrying ArGt-3	This study
pET-Duet-pgm2.galU	pET-Duet vector carrying pgm2.galU	This study
piBR181-pgm2.galU.UGT78K1	piBR181 vector carrying pgm2.galU.UGT78K1	This study
piBR181-glf.glk.pgm2.galU.UGT78K1	piBR181 vector carrying glf.glk.pgm2.galU.UGT78K1	This study
pCDF-Duet-tgs.dh	pCDF-Duet vector carrying tgs.dh	Simkhada et al. [31]
pACYC-Duet-ep.kr	pACYC-Duet vector carrying ep.kr	Simkhada et al. [31]
piBR181-pgm2.galU	piBR181 vector carrying pgm2.galU	This study
piBR181-tgs.dh.ep.kr	piBR181 vector carrying tgs.dh.ep.kr	This study
PiBR181-tgs.dh.ep.kr.pgm2	piBR181 vector carrying tgs.dh.ep.kr.pgm2.galU	This study
piBR181-tgs.dh.ep.kr.pgm2. glf.glk	piBR181 vector carrying tgs.dh.ep.kr.pgm2.galU.glf.glk	This study
piBR181-glf	piBR181 carrying glf from *Zymomonas mobilis* ZM4	This study
piBR181-glk	piBR181 carrying glk gene *Zymomonas mobilis* ZM4	This study

Strains	Description	Strain number	Source/references
*Escherichia coli* XL-1 blue (MRF’)	General cloning host		Stratagene, USA
*E. coli* BL21 (DE3)	*ompT hsdT hsdS* (r_B_-m_B_-) gal (DE3)		Novagen
*E. coli* BL21 (DE3)/piBR181-UGT78K1	BL21(DE3) carrying piBR181-UGT78K1 from *Glycin max* (L.) Merr	S_1_	This study
*E. coli* BL21 (DE3) pET-Duet-pgm2.galU piBR181.UGT78K1	BL21(DE3) carrying pET-Duet-pgm2.galU and piBR181-UGT78K1	S_2_	This study
*E. coli* BL21 piBR181-pgm2.galU.UGT78K1	BL21(DE3) carrying piBR181-pgm2.galU.UGT78K1	S_3_	This study
*E. coli* BL21 piBR181-glf.glk.pgm2.galU.UGT78K1	BL21(DE3) carrying piBR181-glf.glk.pgm2.galU.UGT78K1	S_4_	This study
*E. coli* BL21 (DE3) pET32a-ArGt-3	BL21 (DE3) pET32 + vector carrying ArGt-3 from *Arabidopsis thaliana*	S_5_	This study
*E. coli* BL21 (DE3) pCDF-Duet-tgs.dh. pACYC-Duet-ep.kr. pET32a-ArGt-3	BL21 (DE3) carrying pCDF-Duet-tgs.dh. pACYC-Duet-ep.kr. pET32a-ArGt-3	S_6_	This study
*E. coli* BL21 (DE3) pCDF-Duet-tgs.dh. pACYC-Duet-ep.kr. piBR181.pgm2. pET32a-ArGt-3	BL21 (DE3) carrying pCDF-Duet-tgs.dh. pACYC-Duet-ep.kr. piBR181.pgm2. pET32a-ArGt-3	S_7_	This study
*E. coli* BL21 (DE3) piBR181.tgs.dh.ep.kr.pgm2. pET32a-ArGt-3	BL21 (DE3) carrying piBR181.tgs.dh.ep.kr.pgm2. pET32a-ArGt-3	S_8_	This study
*E. coli* BL21 (DE3) piBR181.tgs.dh.ep.kr.glf.glk.pgm2. pET32a-ArGt-3	BL21 (DE3) carrying piBR181.tgs.dh.ep.kr.glf.glk.pgm2. pET32a-ArGt-3	S_9_	This study

### Nucleic acid manipulation and construction of vectors

All the sugar pathway genes were overexpressed into *E. coli* BL21 (DE3) for specific sugar cassettes construction. To enhance the cytoplasmic pool of UDP-glucose in cell cytosol, the genes for glucokinase (*glk*—*Zymomonas mobilisi*, GenBank accession no. AE008692.2), phosphoglucomutase (*pgm2*—*Bacillus licheniformis* DSM 13, GenBank accession no. YP_006712377.1), glucose 1-phosphate uridylyltransferase (*galU*—*E. coli* K-12, GenBank accession no. CP001509.3) and glucosyltransferases (*UGT78K1*—*G. max* (L) Merr. (Black Soybean), GenBank accession no. ADC96620.1) including a glucose facilitator diffusion protein (*glf*—*Z. mobilisi*, GenBank accession no. AE008692.2) provided by Dr. Stephanie Bringer-Meyer (Institute of Bio- and Geoscience, Germany) [32] were cloned in pIBR181 vector to construct the glucose cassette containing the glucosylation system. *glf*, *glk*, *pgm2* including glucose 1-phosphate thymidylyltransferase (*tgs*—*E. coli* K-12, GenBank accession no. YP_490281.1), TDP-glucose 4, 6-dehydratase (*dh*—*Salmonella enterica* LT2, GenBank accession no. NC_003197.1), TDP-4-keto-6-deoxyglucose 3, 5-epimerase and TDP-glucose 4-ketoreductase (*ep* and *kr*—*Streptomyces antibioticus* Tu99 with GenBank AAF59933.1 and AF237894.1, respectively) were cloned in the same pIBR181 vector under individual T7 promoter. Using the primer pairs of *Xba*I ‘F’ and *Hin*dIII ‘R’, all the genes involved in UDP-glucose and TDP-rhamnose cassettes were PCR amplified from their respective sources and manipulated into the pGEM®-T easy vector. The nucleotide sequences were later confirmed by sequencing. Three genes *dh*, *ep* and *kr* were PCR amplified from the previously reported vector constructs [31] for cloning in pIBR181. Finally, the sugar pathway genes, including glucosyltransferase (*UGT78K1*), containing terminal *Xba*I/*Hind*III restriction sites were individually cloned into the endonuclease digested vector piBR181 at *Spe*I/*Hind*III sites for the final construct. The vector piBR181 is a genetic circuit containing 181-bp double stranded DNA and includes computationally designed bio-parts between the *Bgl*II and *Xho*I restriction site and the *Spe*I/*Hind*III cloning site for single ligation. *Bam*HI/*Xho*I restriction sites are located before the promoter and after the transcription termination to facilitate multiple assemblies of the sugar pathway genes [26]. Unfortunately, rhamnosyltransferase (ArGt-3, *A. thaliana*, GenBank accession no. AF360160) [31] could not be cloned into piBR181 because of the five enzymes restriction. So, this glycosyltransferase was cloned into pET32a(+) vector in *EcoR*I/*Xho*I sites under the same promoter (T7) and plasmid copy number as pIBR181.

### Substrate inhibition and optimization

Bacterial strains harboring only GTs, S_1_ and S_5_ of the same cell density were used for the substrate inhibition test. Different concentrations of fisetin (0.2 mM, 0.3 mM, 0.4 μM, 0.6 mM, 0.8 mM, 1.0 mM) dissolved in dimethylsulfoxide (DMSO) was used to bio-transform in fed batch 50 mL culture volume. At equal incubation time intervals (i.e. 6 h intervals for 24 h and 12 h intervals for 60 h) but the data was considered of 48 h for analyses, 1 mL samples were taken from each flask and centrifuged at 12,000 rpm for 10 min. The supernatants were extracted using a double volume of ethyl acetate while the cell pellets were re-suspended in water and the cell densities measured by spectrophotometer at 600 nm ultraviolet (UV) absorbance. The ethyl acetate fractions were dried and re-suspended into 100 μL methanol and subjected to photo diode array connected high performance liquid chromatography (HPLC–PDA) analyses. The peak area was used to calculate the total conversion of substrate into glycoside product over cell growth in reference to the standard curve of fisetin.

### Glucose supplement and optimization

Recombinant strains S_1_ and S_5_ were also considered for glucose supplementation and optimization in a biotransformation reaction. These strains were grown under identical conditions, keeping *E. coli* BL21 (DE3) as a control strain for growth measurement. The samples used for HPLC–PDA analysis and cell density measurement were prepared as mentioned above. Optimized fisetin substrate concentration (0.3 mM) was supplemented in the cultures with three different concentrations of glucose (5, 10 and 15%) to determine the optimum glucose concentration for biotransformation. Finally, HPLC–PDA analysis was carried out and conversion of the substrate into product was verified over incubation time intervals according to the cell growth.

### Bioconversion and Scale-up whole-cell biotransformation

*E. coli* strains S_1_ and S_5_ were used for whole cell bio-catalysis assay. These strains were grown on LB medium supplemented with kanamycin (50 μg/mL) and ampicillin (100 μg/mL) antibiotics, respectively. The cultures were induced with 0.5 mM isopropyl-1-thio-β-d-galactopyranoside (IPTG) when the cells’ OD_600 nm_ reached 0.5–0.7 while incubating at 37°C. The cultures were incubated at 20°C for 20 h for functional expression of protein. After 20 h of incubation at 20°C, cultures were fed with 0.3 mM fisetin and incubated for 48 h to produce fisetin-3-*O*-glucoside and fisetin 3-*O*-rhamnoside from the respective strains. Identical conditions were used for comparison of the biotransformation of fisetin using other strains (S_1_–S_9_) to determine the best strain for large-scale production of flavonol glycosides. The biotransformation of other flavonols (quercetin, kaempferol, myricetin, and morin) was also carried out under identical conditions.

For the lab scale biotransformation of fisetin in 3 L fermentor using the best optimized strains (S_4_ and S_9_), identical conditions of pH, temperature (25°C) and dissolved oxygen were used as reported previously [30]. Sterile glucose (10% final concentration) was supplemented to the fermentor at every 1 h intervals after addition of fisetin to the induced cultures up to 36 h. The samples were taken at every 12 h intervals for the HPLC–PDA analysis up to 60 h after initial addition of fisetin (300 mg–0.35 mM) to the grown and induced cultures of S_4_ and S_9_. The same amount of fisetin was added after 12 h and 24 h of first addition of fisetin as it was completely converted at 12 h intervals.

### Product analysis and quantification

The culture extracts dissolved in methanol were directly analyzed by reverse-phase HPLC–PDA connected with C_18_ column (Mightysil RP-18 GP (4.6 × 250 mm, 5 μm) at 320 nm using binary conditions of H_2_O (0.05% trifluroacetic acid buffer) and 100% acetonitrile (ACN) at a flow rate of 1 ml/min for 30 min. The ACN concentrations were: 10% (0–2 min), 70% (20–24 min), 100% (24–28 min), and 50% (28–30). For quantification of flavonoids, a calibration curve of authentic fisetin was created using 10, 25, 50, 100 and 200 μg/mL concentrations. The exact mass of the compounds were analyzed by liquid chromatography quadrupole time-of-flight electrospray ionization mass spectrometry LC-QTOF-ESI/MS [ACQUITY (UPLC, Waters, Mil-ford, MA)-SYNAPT G2-S (Waters)] in the positive ion mode.

## Additional files

Additional file 1:
**Figures S1**. Construction of a recombinant glucose cassette using piBR181 mono-cistronic vector. **Figure S2**. Construction of an efficient rhamnosylation system using piBR181 mono-cistronic vector. The figure showing vector map containing cloning sites in a single circuit. **Figure S3**. HPLC-PDA analyses of other flavonols (kaempferol, myricetin, quercetin and morin). **Figure S4**. High resolution mass analyses confirmed flavonol glycosides.

## Abbreviations

TDP: thymidine diphosphate
UDP: uridine diphosphate
NDP: nucleotide diphosphate
HPLC: high performance liquid chromatography
PDA: photo diode array
LC: liquid chromatography
QTOF: quadrupole time-of-flight
ESI/MS: electrospray ionization mass spectrometry
DMSO: dimethyl sulfoxide
IPTG: isopropyl β-d-1-thiogalactopyranoside
ACN: acetonitrile
TFA: trifluroacetic acid

## References

[1] Xiao J, Muzashvili TS, Georgiev MI (2014). Advances in the biotechnological glycosylation of valuable flavonoids. Biotechnol Adv.

[2] Mouri C, Mozaffarian V, Zhang X, Laursen R (2014). Characterization of flavonols in plants used for textile dyeing and the significance of flavonol conjugates. Dyes Pigm.

[3] Perez-Vizcaino F, Duarte J (2010). Flavonols and cardiovascular disease. Mol Aspects Med.

[4] Kim H, Bartley GE, Arvik T, Lipson R, Nah SY, Seo K, Yokoyama W (2014). Dietary supplementation of chardonnay grape seed flour reduces plasma cholesterol concentration, hepatic steatosis, and abdominal fat content in high-fat diet-induced obese hamsters. J Agric Food Chem.

[5] Escriche I, Kadar M, Juan-Borrás M, Domenech E (2014). Suitability of antioxidant capacity flavonoids and phenolic acids for floral authentication of honey. Impact of industrial thermal treatment. Food Chem.

[6] Liu YJ, Zhan J, Liu XL, Wang Y, Jia J, He QQ (2014). Dietary flavonoids intake and risk of type 2 diabetes: a meta-analysis of prospective cohort studies. Clin Nutr.

[7] Liao XL, Luo JG, Kong LY (2013). Flavonoids from *Millettia nitida var*. Hirsutissima with their anti-coagulative activities and inhibitory effects on NO production. J Nat Med.

[8] Wang X (2009). Structure, mechanism and engineering of plant natural product glycosyltransferases. FEBS Lett.

[9] Bartmańska A, Tronina T, Popłoński J, Huszcza E (2013). Biotransformation’s of prenylated hop flavonoids for drug discovery and production. Curr Drug Metab.

[10] Wang A, Zhang F, Huang L, Yin X, Li H, Wang Q, Zeng Z, Xie T (2010). New progress in biocatalysis and biotransformation of flavonoids. J Med Plants Res.

[11] Kamionka M (2011). Engineering of therapeutic proteins production in *Escherichia coli*. Curr Pharm Biotechnol.

[12] Chen X, Zhou L, Tian K, Kumar A, Singh S, Prior BA, Wang Z (2013). Metabolic engineering of *Escherichia coli*: a sustainable industrial platform for bio-based chemical production. Biotechnol Adv.

[13] Fujii T, Narikawa T, Sumisa F, Arisawa A, Takeda K, Kato J (2006). Production of alpha, omega-alkanediols using *Escherichia coli* expressing a cytochrome P450 from Acinetobacter OC4. Biosci Biotechnol Biochem.

[14] Lee SK, Chou H, Ham TS, Lee TS, Keasling JD (2008). Metabolic engineering of microorganisms for biofuels production:from bugs to synthetic biology to fuels. Curr Opin Biotechnol.

[15] Alper H, Stephanopoulos G (2009). Engineering for biofuels: exploiting innate microbial capacity or importing biosynthetic potential. Nat Rev Microbiol.

[16] Kwiecien I, Szopa A, Madej K, Ekjert H (2013). Arbutin production via biotransformation of hydroquinone in in vitro cultures of aromia melanocarpa (Michx) Elliott. Acta Biochim Pol.

[17] Yende SR, Harle UN, Chugule BB (2014). Therapeutic potential and health benefits of Sargassum species. Pharmacogn Rev.

[18] Alonso-Gutierrez J, Chan R, Batth TS, Adams PD, Keasling JD, Petzold CJ, Lee TS (2013). Metabolic engineering of *Escherichia coli* for limonene and perillyl alcohol production. Metab Eng.

[19] Lim CG, Fowler ZL, Hueller T, Schaffer S, Koffas MA (2011). High-yield resveratrol production in engineered *Escherichia coli*. Appl Environ Microbiol.

[20] Negrete A, Ng WI, Shiloach J (2010) Glucose uptake regulation in *E. coli* by the small RNA SgrS: comparative analysis of *E. coli* K-12 (JM109 and MG 1655) and *E. coli* B (BL21). Microb Cell Fact 9:75 10.1186/1475-2859-9-75 PMC2955591 20920177

[21] Shiloach J, Rinas U (2009) Glucose and acetate metabolism in *E. coli*–System level analysis and biotechnological applications in protein production process. In: Lee SY (ed) System biology and biotechnology of *Escherichia coli*. Springer Science + Business Media B.V., USA pp 377–400

[22] Miyahisa I, Funa N, Ohnishi Y, Martens S, Moriguchi T (2006). Sueharu: combinatorial biosynthesis of flavones and flavonols in *Escherichia coli*. Appl Microbiol Biotechnol.

[23] Horinouchi S (2009). Combinatorial biosynthesis of plant medicinal polyketides by microorganisms.

[24] He W, Fu L, Li G, Andrew Jones J, Linhardt RJ, Koffas M (2015). Production of chondroitin in metabolically engineered *E. coli*. Metab Eng.

[25] Zhao S, Jones JA, Lachance DM, Bhan N, Khalidi O, Vankataraman S, Wang Z, Koffas MA (2014). Improvement of catechin production in *Escherichia coli* through combinatorial metabolic engineering. Metab Eng.

[26] Chaudhary AK, Park JW, Yoon YJ, Kim BG, Sohng JK (2013). Re-engineering of genetic circuit for 2-deoxystreptamine (2-DOS) biosynthesis in *Escherichia coli* BL21 (DE3). Biotechnol Lett.

[27] Sichwart S, Hetzler S, Broker D, Steinbuchel A (2011). Extension of the substrate utilization range of *Ralstonia eutropha* strain H16 by metabolic engineering to include mannose and glucose. Appl Environ Microbiol.

[28] Tozakidis IE, Sichwart S, Teese MG, Jose J (2014). Autotransporter mediated esterase display on *Zymomonas mobilis* and *Zymobacter palmae*. J Biotechnol.

[29] Jones P, Messner B, Nakajima J, Schaffner AR, Saito K (2003). UGT73C6 and UGT78D1, glycosyltransferases involved in flavonol glycoside biosynthesis in *Arabidopsis thaliana*. J Biol Chem.

[30] Pandey RP, Malla S, Simkhada D, Kim BG, Sohng JK (2013). Production of 3-*O*-xylosyl quercetin in *Escherichia coli*. Appl Microbiol Biotechnol.

[31] Simkhada D, Lee HC, Sohng JK (2010). Genetic engineering approach for the production of rhamnosyl and allosyl flavonoids from *Escherichia coli*. Biotechnol Bioeng.

[32] Siedler S, Bringer S, Blank LM, Bott M (2012). Engineering yield and rate of reductive biotransformation in *Escherichia coli* by partial cyclization of the pentose phosphate pathway and PTS-independent glucose transport. Appl Microbiol Biotechnol.

[33] Gabor M, Eperjessy E (1966). Antibacterial effect of fisetin and fisetidin. Nature.

[34] Malla S, Pandey RP, Kim BG, Sohng JK (2013). Regiospecific modifications of naringenin for astragalin production in *Escherichia coli*. Biotechnol Bioeng.

[35] Lim HN, Lee Y, Hussein R (2011). Fundamental relationship between operon organization and gene expression. Proc Natl Acad Sci USA.

[36] Xu P, Vansiri A, Bhan N, Koffas MA (2012). ePathBrick: a synthetic biology platform for engineering metabolic pathways in *E. coli*. ACS Synth Biol.

[37] Ferrari R, Rivetti C, Dieci G (2004). Transcription reinitiation properties of bacteriophage T_7_ RNA polymerase. Biochem Biophys Res Commun.

[38] Thuan NH, Pandey RP, Thuy TT, Park JW, Sohng JK (2013). Improvement of region-specific production of myricetin-3-*O*-α-l-rhamnoside in engineered *Escherichia coli*. Appl Biochem Biotechnol.

[39] Kim BG, Kim HJ, Ahn JH (2012). Production of bioactive flavonol rhamnosides by expression of plant genes in *Escherichia coli*. J Agric Food Chem.

[40] Yoon JA, Kim BG, Lee WJ, Lim Y, Chong Y, Ahn JH (2012). Production of a novel quercetin glycoside through metabolic engineering of *Escherichia coli*. Appl Environ Microbiol.

[41] Mao Z, Shin HD, Chen RR (2006). Engineering the *E. coli* UDP-glucose synthesis pathway for oligosaccharide synthesis. Biotechnol Prog.

[42] Aw R, Polizzi MK (2013). Can too many copies spoil broth?. Microb Cell Fact.

[43] Xu P, Koffas MA (2013). Assembly of multi-gene pathways and combinatorial pathway libraries through ePathBrick vectors. Methods Mol Biol.

[44] Na D, Kim TY, Lee SY (2010). Construction and optimization of synthetic pathways in metabolic engineering. Curr Opin Microbiol.

[45] Stephanopoulos G (2012). Synthetic biology and metabolic engineering. ACS Synth Biol.

[46] Du S, Li C, Wang Y, Liu C, Ren D, Li Y (2012). Construction and evaluation of a new triple-gene expression cassette vaccinia virus shuttle vector. J Virol Methods.

[47] Li M, Wang J, Geng Y, Wang Q, Qi Q (2012). A strategy of gene overexpression based on tandem repetitive promoters in *Escherichia coli*. Microb Cell Fact.

