# Supplementary material for: Synthetic sugar cassettes for the efficient production of flavonol glycosides in Escherichia coli
Source: Microb Cell Fact. 2015 Jun 9;14:76. doi: 10.1186/s12934-015-0261-1 (PMC4459062; doi:10.1186/s12934-015-0261-1)

*Microbial Cell Factories*

**Synthetic sugar cassettes for the efficient production of flavonol glycosides in**

***Escherichia coli***

Prakash Parajuli^†^

[parajuli1985@gmail.com](mailto:parajuli1985@gmail.com)

Ramesh Prasad Pandey

[pandey@sunmoon.ac.kr](mailto:pandey@sunmoon.ac.kr)

Nguyen Thi Huyen Trang

[nguyenhuyentrang0512@gmail.com](mailto:nguyenhuyentrang0512@gmail.com)

Amit Kumar Chaudhary

[amitkumarchaudhary3@gmail.com](mailto:amitkumarchaudhary3@gmail.com)

Jae Kyung Sohng*

[sohng@sunmoon.ac.kr](mailto:sohng@sunmoon.ac.kr)

***Correspondence author:** Institute of Biomolecule Reconstruction, Department of BT-Convergent Pharmaceutical Engineering, Sun Moon University, 70 Sunmoon-ro 221, Tangjeong-myeon, Asan-si, Chungnam 336-708, Republic of Korea.

Tel: +82(41)530-2246 Fax: +82(41)544-2919

**Legends for table**

**Table S1.** List of primers used in this study

**Figure legends**

**Figure S1.** Construction of a recombinant glucose cassette using piBR181 mono-cistronic vector. The final model construct piBR181-glf.glk.pgm2.galU.UGT78K1 contains NDP-sugar biosynthesis pathway genes; glucokinase (*glk*), phosphoglucomutase (*pgm2*), glucose 1-phosphate uridylyltransferase (*galU*) and flavonoid 3-*O*-glycosyltransferase (*UGT78K1*) including glucose facilitator diffusion protein (*glf*). The figure showing, all of the genetic circuits were assembled into a single vector based on the strategy reported previously.

**Figure S2.** Construction of an efficient rhamnosylation system using piBR181 mono-cistronic vector. The figure showing vector map containing cloning sites in a single circuit. To increase the TDP-L-rhamnose pool in cytosol of the cell, all genes involved in NDP-sugar biosynthesis pathway; glucokinase (*glk*), phosphoglucomutase (*pgm2*), TDP-glucose synthase (*tgs*), TDP-glucose 4,6-dehydratase (*dh*), TDP-4-keto-6-deoxyglucose 3,5-epimerase (*epi*) and TDP-glucose 4-ketoreductase (*kr*) were cloned and assembled into a single vector piBR181 including glucose facilitator protein (*glf*). The figure showing, NDP-sugar biosynthesis genes are assembled into single vector and glycosyltransferases (*ArGt-3*) is cloned into pET32(a)+ in order to make compactible strain with NDP-rhamnose biosynthesis system.

**Figure S3.** HPLC-PDA analyses of other flavonols (kaempferol, myricetin, quercetin and morin) in strain S_4_ and S_9_ at 48 h incubation supplemented with 10% glucose. (**A**) Production of flavonols 3-*O*-rhamnoside from the strain S_9_ harboring deoxy sugar cassette catalyzed by ArGt-3. (**B**) Production of flavonols 3-*O*-glucoside from the strain S_4_ harboring UDP-glucose cassette catalyzed by UGT78K1. Dark circle indicates the remaining substrate in respective retention time while shaded area shows the production of glycosides.

**Figure S4.** High resolution mass analyses confirmed each flavonol glycosides. (**A**) Structure of each flavonols 3-*O*-rhamnoside and respective mass spectrums (**B**) structure of each flavonol 3-*O*-glucoside and respective mass spectrums. Mass spectrums were detected as [kaempferol 3-*O*-rhamnoside + M] ^+^ *m/z* ~ 433.1132, [myricetin 3-*O*-rhamnoside + M] ^+^ *m/z* ~ 465.1035, [quercetin 3-*O*-rhamnoside + M] ^+^ *m/z* ~ 449.1078 and [morin 3-*O*-rhamnoside + M] ^+^ *m/z* ~ 449.1078 where as flavonols glucoside were [kaempferol 3-*O*-glucoside + M] ^+^ *m/z* ~ 449.1075, [myricetin 3-*O*-glucoside + Na] ^+^ *m/z* ~ 481.0985, [quercetin 3-*O*-glucoside + M] ^+^ *m/z* ~ 465.1028 and [morin 3-*O*-glucoside + M] ^+^ *m/z* ~ 465.1044.

**Table S1.**

| Primers | Oligonucleotide sequences (5’-3’) | Restriction sites |
| --- | --- | --- |
| glf-F | TCTAGAATGAGTTCTGAAAGTAGTCAGGGTCTA | *Xba*I |
| glf-R | AAGCTT CTACTTCTGGGAGCGCCACATCTCCTC | *Hind*III |
| glk-F | TCTAGAATGGAAATTGTTGCGATTGACATCGGT | *Xba*I |
| glk-R | AAGCTTTTATTCAACTTCAGAATATTTGTTGGC | *Hind*III |
| pgm2-F | TCTAGAATGAGCTGGAGAACGAGCTATGAACGC | *Xba*I |
| pgm2-R | AAGCTTTTACGAATTTGAGGTCGCTTTTACAAT | *Hind*III |
| galU-F | TCTAGAATGGCTGCCATTAATACGAAAGTCAAA | *Xba*I |
| galU-R | AAGCTTTTACTTCTTAATGCCCATCTCTTCTTC | *Hind*III |
| UGT78K1-F | TCTAGA ATGGATCATCAAAACAAACAC | *Xba*I |
| UGT78K1-R | AAGCTTTTAAGACCTAGAAATTACTTC | *Hind*III |
| tgs-F | TCTAGAATGAAAATGCGTAAAGGTATT | *Xba*I |
| tgs-R | AAGCTTTTAATTTGAATCCTTCGTCAT | *Hind*III |
| dh-F | TCTAGAGTGAAGATACTTATTACTGGC | *Xba*I |
| dh-R | AAGCTTTTACTGGCGTCCTTCATAGTT | *Hind*III |
| epi-F | TCTAGAATGGAGTTACTCGACGTCGAC | *Xba*I |
| epi-R | AAGCTTTCACCGGGCCGGTCCCACGCC | *Hind*III |
| kr-F | TCTAGAATGAGATGGCTGATCACCGGC | *Xba*I |
| kr-R | AAGCTTTCATGCTGCTCCTCGCCGGGT | *Hind*III |
| glf-F | TCTAGA*ATGAGTTCTGAAAGTAGTCAGGGT* | *XbaI* |
| glf-R | AAGCTTCTACTTCTGGGAGCGCCACATCTC | *HindIII* |
| glk-F | TCTAGAATGGAAATTGTTGCGATTGACATC | *XbaI* |
| glk.R | AAGCTTTTAAAAAATATTATTCAACTTCAG | *HindIII* |

**Figure S1.**





**Figure S2.**





**Figure S3.**


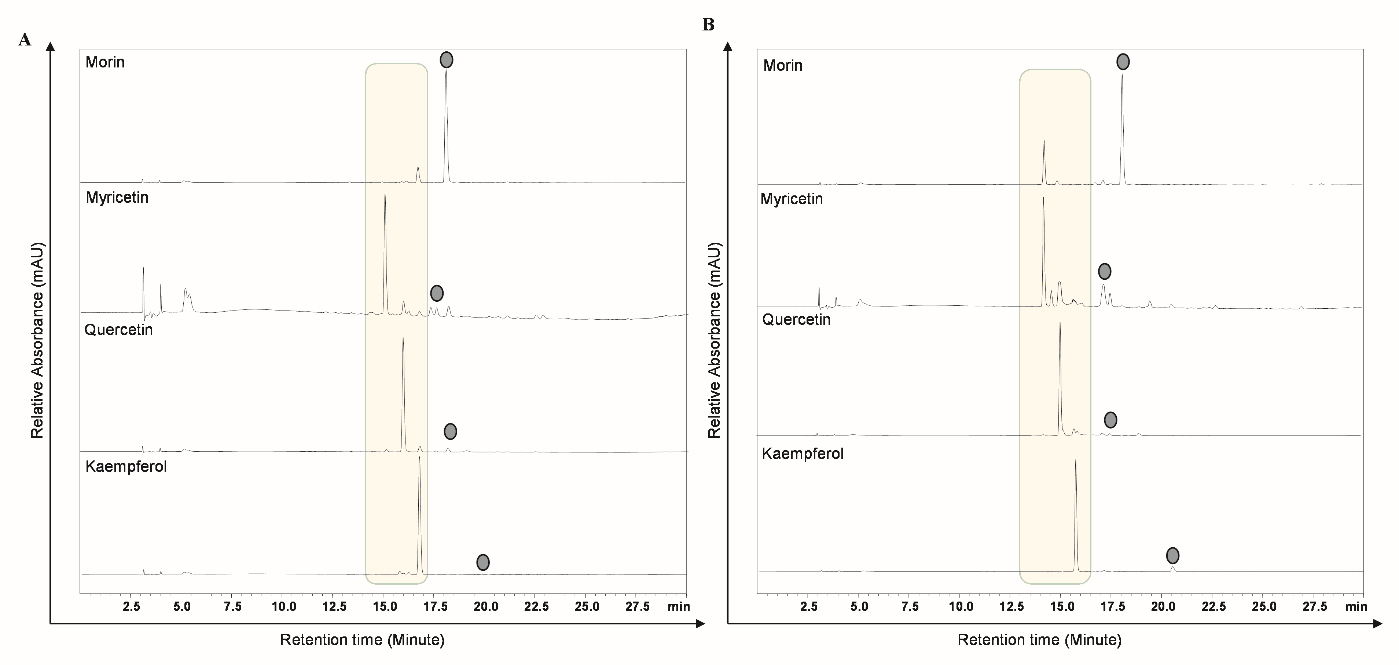


**Figure S4.**


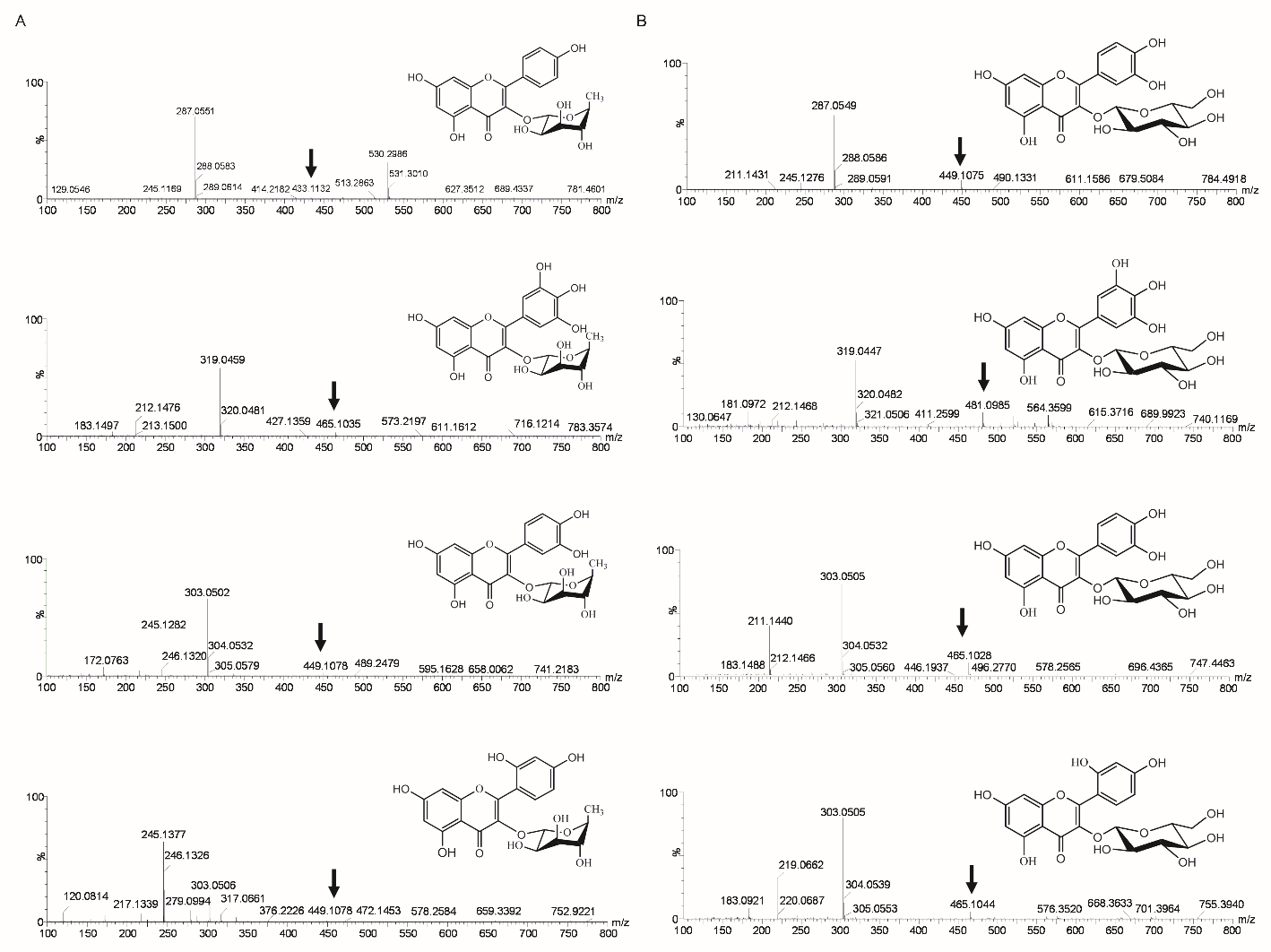

Supplement: Additional file 1: — Figures S1. Construction of a recombinant glucose cassette using piBR181 mono-cistronic vector. Figure S2. Construction of an efficient rhamnosylation system using piBR181 mono-cistronic vector. The figure showing vector map containing cloning sites in a single circuit. Figure S3. HPLC-PDA analyses of other flavonols (kaempferol, myricetin, quercetin and morin). Figure S4. High resolution mass analyses confirmed flavonol glycosides. [file 12934_2015_261_MOESM1_ESM.docx]
